# Inertial sensor data of horses from four breeds at walk and trot in hand on a straight line

**DOI:** 10.1016/j.dib.2024.110764

**Published:** 2024-07-22

**Authors:** Annik Imogen Gmel, Eyrún Halla Haraldsdóttir, Filipe Serra-Bragança, Luis P Lamas, Teresa V Rosa, Monika Stefaniuk-Szmukier, Weronika Klecel, Markus Neuditschko, Michael A Weishaupt

**Affiliations:** a Agroscope, Animal GenoPhenomics, Route de la Tioleyre 4, 1725 Posieux, Switzerland; b Equine Department, Vetsuisse Faculty, University of Zurich, Winterthurerstrasse 260, 8057 Zurich, Switzerland; c Department of Clinical Sciences, Faculty of Veterinary Medicine, Utrecht University, Yalelaan 114, 3584 CM, Utrecht, the Netherlands; d CIISA, Faculdade de Medicina Veterináriada Universidade de Lisboa, Lisboa, Portugal; e National Research Institute of Animal Production, University of Agriculture in Krakow, Krakow, Poland; f Department of Animal Genetics and Conservation, Institute of Animal Sciences, Warsaw University of Life Sciences, Warsaw, Poland

**Keywords:** Equine, Locomotion, Gait quality, Stride length, Protraction, Retraction

## Abstract

Horses have been used and bred for centuries for their movements. However, specific breeds are expected to have different movement capabilities. We have measured 425 horses from four different breeds at walk and trot on a straight line using an inertial measurement unit (IMU) system (EquiMoves®). This article describes how the data was collected, filtered and analysed to provide a useable dataset of 28 movement variables. It provides a full protocol for field measurements and requirements for adequate trials for analysis. Intra-class correlation coefficient estimates are also provided to assess repeatability of the measurements.

Specifications Table

**Table:** 

Subject	Veterinary Science
Specific subject area	Kinematic, time and space variables for 425 sound horses of four different breed measured with the EquiMoves® inertial sensor system at the walk and trot, on a hard or soft surface.
Data format	Analyzed, Filtered
Type of data	csv table containing extracted numerical data from EquiMoves® software
Data collection	Each horse was equipped with seven IMU sensors (Promove -mini) from the EquiMoves® system (one on the poll, on the withers, on the croup, and one on each limb) and walked and trotted on a straight line over 25–46 m. We selected the runs at each gait for which the horse was in a steady-state movement, concentrated, looking ahead, not changing gait, tossing its head, jumping or kicking. Data collection was done with EquiMoves® motion processing software (version 0.0.211001), which is equipped with algorithms for filtering, stride splitting and gait detection along with computing relevant upper-and lower body variables. The processed data from the EquiMoves® was exported to Matlab, where the usable measurements were extracted and further post-processed, using custom made scripts, for batch analysis of the data.
Data source location	Institution: University of Zurich City/town/region: Zurich Country: Switzerland
Data accessibility	Repository name: Mendeley Data Data identification number: 10.17632/c24tf9332k.3 Direct URL to data: https://data.mendeley.com/datasets/c24tf9332k/2

## 1 Value of the Data

• Large publicly available dataset of IMU motion data of horses walking and trotting in hand on a straight line
• Provides valuable information on how horses move according to age, sex, height and breed
• Can be used by any researcher interested in locomotion patterns in horses. We are providing our complete methodology of data collection, so that researchers can create comparative datasets in prospective studies
• Can be used to establish reference populations for breed standards or provide comparative values when studying additional breeds
• Can be used to define an acceptable level of asymmetry in owner-sound horse (before it becomes clinically relevant),
• Can be used to estimate heritabilities, breeding values and perform genome-wide association studies

## 2 Data Description

The dataset consists of one csv file with 46 variables (columns) and 916 rows (one header and 915 measurements). The variables and their abbreviations are described in Table 1. After the first four columns (the individual identifier, the gait, the mean speed and the device used to measure speed), the following 28 columns are measured variables. The last columns are providing relevant context to the measured horse (breed, age and sex of the horse, withers height, limb length, shoeing status), as well as information on the location, dates of measurement and the handlers, which were the same for walk and trot.

**Table 1:** Definition of abbreviations in the data.

Abbreviation	Definition
HorseID	Individual horse identifier
Gait	Gait at which the horse was measured (walk or trot)
Speed	Mean speed over all selected trials per gait [m/s]
Speed_mes	Measurement system providing the timing to calculate mean speed (Stopwatch, Freelap or EquiMoves)
MaxPro_lf	Maximal protraction angle of the left front limb [deg]
MaxPro_rf	Maximal protraction angle of the right front limb [deg]
MaxPro_lh	Maximal protraction angle of the left hind limb [deg]
MaxPro_rh	Maximal protraction angle of the right hind limb [deg]
MaxRet_lf	Maximal retraction angle of the left front limb [deg]
MaxRet_rf	Maximal retraction angle of the right front limb [deg]
MaxRet_lh	Maximal retraction angle of the left hind limb [deg]
MaxRet_rh	Maximal retraction angle of the right hind limb [deg]
MaxAbd_lf	Maximal abduction angle of the left front limb [deg]
MaxAbd_rf	Maximal abduction angle of the right front limb [deg]
MaxAbd_lh	Maximal abduction angle of the left hind limb [deg]
MaxAbd_rh	Maximal abduction angle of the right hind limb [deg]
MaxAdd_lf	Maximal adduction angle of the left front limb [deg]
MaxAdd_rf	Maximal adduction angle of the right front limb [deg]
MaxAdd_lh	Maximal adduction angle of the left hind limb [deg]
MaxAdd_rh	Maximal adduction angle of the right hind limb [deg]
MeanStD_lf	Mean stance duration of the left front limb [s]
MeanStD_rf	Mean stance duration of the right front limb [s]
MeanStD_lh	Mean stance duration of the left hind limb [s]
MeanStD_rh	Mean stance duration of the right hind limb [s]
MeanSD	Mean stride duration [s]
MeanSF	Mean stride frequency [strides/sec or Hz]
ROMWithers	Mean vertical range of motion of the withers [mm]
Susp_L	Mean left suspension duration at trot. Time of hoof-off of the left front or right hind limb (whichever limb that has a later hoof-off) to the time of hoof on of the right front or the left hind limb (whichever limb that has an earlier hoof-on). Set to NA at walk [s]
Susp_R	Mean right suspension duration at trot. Time of hoof-off of the right front or left hind limb (whichever limb that has a later hoof-off) to time of hoof-on of the left front or right hind limb (whichever limb that has an earlier hoof-on). Set to NA at walk [s]
Stride_count	Number of strides taken into account for the mean
MeanSL	Mean stride length [m]. Mean speed × stride duration.
ROMWithers_rel	Mean vertical range of motion of the withers relative to the withers’ height of the horse
Breed	Breed (FM=Franches-Montagnes, WB=European Warmblood, AR=Purebred Arabian, LUS=Lusitano)
Sex	Sex (g=gelding, m=mare, s=stallion)
YOB	Year of birth
YOM	Year of measurement
LL	Limb length [cm]
WH	Withers height [cm]
SHOE	whether the horses were shod. 0 = unshod, 1 = shod in front, 2 = fully shod
Ground	Hard ground surface (hard surface = asphalt) or soft ground surface (soft surface = sand arena)
Location	Closest village/city to the measurement site
Date	Date (format DD.MM.YYYY)
Runway_length	Length of the runway [m]
Placer	Person equipping the horse with the EquiMoves system and measuring the limb length
Handler 1	Person at the bridle of the horse, walking and trotting alongside the horse
Handler 2	Person motivating the horse from behind

There were 425 different horses measured in total. Overall, 34 horses were measured on several different days at walk and 29 at trot. The majority of measured horses were Franches-Montagnes horses (FM), followed by Lusitano (LUS), Swiss Warmblood (WB), and Purebred Arabian (AR, Table 2). The horses were between three and 29 years old.

**Table 2:** Summary of available data. Number of individual animals (n), sex (stallions, geldings and mares), median year of birth (YOB), and mean age ± standard deviation.

Breed	n	Sex			YOB	Age (across all measurements)
		s	g	m		
FM	318	120	95	103	2018	5.21 ± 4.88
WB	40	1	13	26	2017	3.00 ± 0.00
LUS	52	45	0	7	2014	9.58 ± 4.83
AR	14	1	0	13	2014	9.20 ± 4.28

### 2.1 Repeatability

We used the repeated measurements to estimate the repeatability of the different parameters using intraclass correlation coefficients at walk and trot (Table 3). At walk, 31 horses (one WB gelding, two AR mares, two LUS and 26 FM stallions) were measured twice and three FM stallions were measured three times. The mean number of repeated measures K was thus equal to 2.09. All horses except the two AR mares were measured on a hard surface. Three horses were not shod, the rest were shod on all four. The horses had the same type of shoeing for every repeated measure. The mean measurement for the horses relied on a mean of 45.92 strides, with a minimum of 20 strides and a maximum of 78.

**Table 3:** Repeatability of the kinematic variables using intra-class coefficients, including the confidence interval at the 95 % level. N is the number of individual horses. K is the mean number of repeated measures.

Variable	Walk (*N* = 34, mean *K* = 2.09)	Trot (*N* = 29, mean *K* = 2.14)
Speed	0.68 [0.46;0.82]	0.42 [0.11;0.67]
MaxPro_lf	0.58 [0.32;0.76]	0.49 [0.19;0.72]
MaxPro_rf	0.56 [0.30;0.75]	0.64 [0.39;0.81]
MaxPro_lh	0.84 [0.71;0.92]	0.73 [0.51;0.86]
MaxPro_rh	0.85 [0.73;0.92]	0.75 [0.55;0.87]
MaxRet_lf	0.91 [0.83;0.95]	0.53 [0.24;0.74]
MaxRet_rf	0.67 [0.44;0.81]	0.61 [0.34;0.79]
MaxRet_lh	0.72 [0.52;0.85]	0.58 [0.30;0.77]
MaxRet_rh	0.74 [0.55;0.86]	0.34 [0.01;0.61]
MaxAbd_lf	0.37 [0.07;0.62]	0.32 [−0.1;0.60]
MaxAbd_rf	0.80 [0.65;0.89]	0.56 [0.28;0.76]
MaxAbd_lh	0.64 [0.40;0.80]	0.64 [0.38;0.81]
MaxAbd_rh	0.74 [0.55;0.86]	0.80 [0.64;0.90]
MaxAdd_lf	0.77 [0.60;0.88]	0.60 [0.34;0.79]
MaxAdd_rf	0.51 [0.23;0.71]	0.71 [0.49;0.85]
MaxAdd_lh	0.52 [0.24;0.72]	0.81 [0.64;0.90]
MaxAdd_rh	0.87 [0.79;0.94]	0.74 [0.53;0.86]
MeanStD_lf	0.61 [0.37;0.78]	0.54 [0.26;0.75]
MeanStD_rf	0.52 [0.24;0.72]	0.39 [0.06;0.65]
MeanStD_lh	0.60 [0.34;0.77]	0.31 [−0.02;0.59]
MeanStD_rh	0.51 [0.23;0.71]	0.35 [0.03;0.62]
MeanSD	0.57 [0.31;0.75]	0.49 [0.18;0.71]
MeanSF	0.55 [0.28;0.74]	0.53 [0.24;0.74]
MeanSL	0.76 [0.59;0.87]	0.44 [0.12;0.68]
ROMWithers	–	0.74 [0.54;0.87]
Susp_L	–	0.49 [0.19;0.72]
Susp_R	–	0.55 [0.26;0.75]
ROMWithers_rel	–	0.69 [0.46;0.84]

At trot, 25 horses (one AR mare, 1 FM gelding and 23 FM stallions) were measured twice and four FM stallions three times (mean *K* = 2.14). The AR mare was measured on the soft surface and the FM horses on the hard surface. The AR mare and FM gelding were not shod, while the FM stallions were shod on all four. As at the walk, the horses had the same type of shoeing for every repeated measure. The mean measurement for the horses relied on a mean of 32.73 strides, with a minimum of 11 strides and a maximum of 57.

## 3 Experimental Design, Materials and Methods

**Fig. 1:**
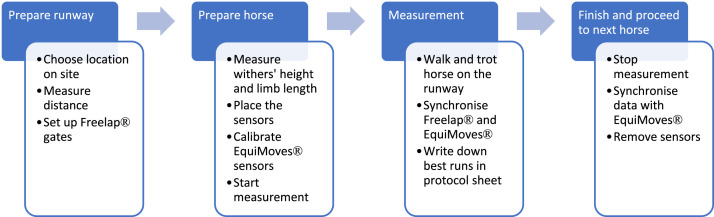
Normal measuring procedure at each location.

For this dataset, we measured 428 horses from four different breeds on a straight line at walk and trot (Fig. 1). They were presented in hand, by experienced handlers. In the initial phase (2020–2021), the focus was to gather data on young Swiss horses presented at the field test at three years old and which would be broken in to ensure that they would tolerate the bridle, girth and boots to which the sensors would be attached. In 2022 and 2023, we expanded the criteria to also include older horses used for breeding when they were available for measurement on farm. The older horses were trained for showing in hand (AR), dressage and equestrian shows (FM and LUS) as well as carriage driving (FM).

During measurements, the horses were also evaluated for potential lameness based on the scale in Weishaupt et al. [1]:

• Grade 1 - subtle lameness: irregularity not visible on every stride at the trot;
• Grade 2 - mild lameness: visible on every stride at the trot;
• Grade 3 - moderate lameness: distinctly visible at the trot where, however, the cadence of the movement is not obviously disturbed.

Measurements of horses which were lame above grade 1 were not included in the dataset.

The speed of the horses was measured separately using electronic timing gates (Freelap®, Freelap SA, Switzerland) as speed could not be measured at all time accurately with the former GPS-node integrated in IMU withers sensor when data collection started in 2020; from 2021 onwards the withers IMU sensor was equipped with a GNSS-node. Electronic timing gates are generally considered the gold standard for measuring speed in human athletes such as sprinters [2,3].

### 3.1 Setup of the runway

**Fig. 2:**
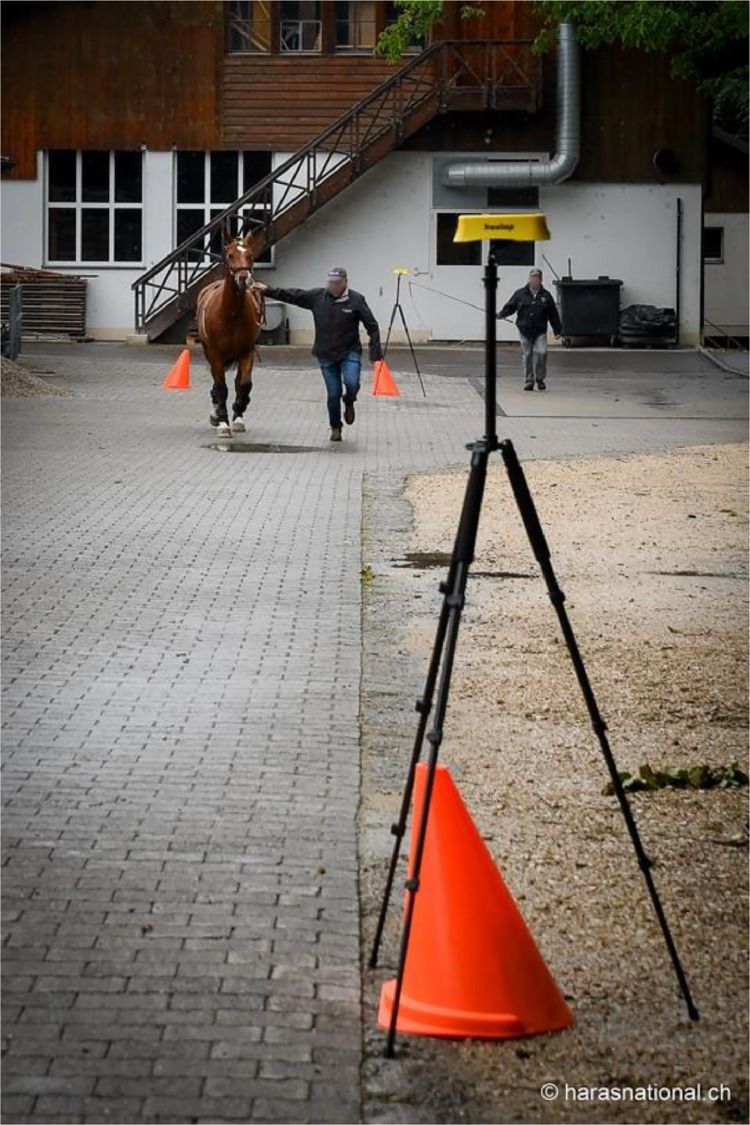
Typical setup of the data collection. A runway of 35 m, a horse equipped with the EquiMoves® system and the Freelap® sensor, trotted by an experienced handler and encouraged by a second person following the horse.

At each location, we delimited a straight runway of 25 to 45.8 m with as little incline as possible outside. Additional space adjacent to the runway (approximately 5 m) was necessary for breaking, turning around and reaccelerating the horse on each end, so that the horse was approximately in a movement steady state over the whole length of the runway. The runway was delimited by two Freelap® gateways (Tx Track Pro) to record the time (Fig. 2).

### 3.2 Preparation of the horse

Withers height was measured on site (AR, LUS) with a measuring stick or was provided by the breeding organizations (WB, FM). Information on withers height was missing from eight horses. From the year 2021, with a few exceptions, limb length was additionally measured using a measuring tape from the bony lateral prominence of the lateral collateral ligament elbow joint down to the laterally palpable joint space of the metacarpophalangeal joint. Limb length was not available for 93 horses.

**Fig. 3:**
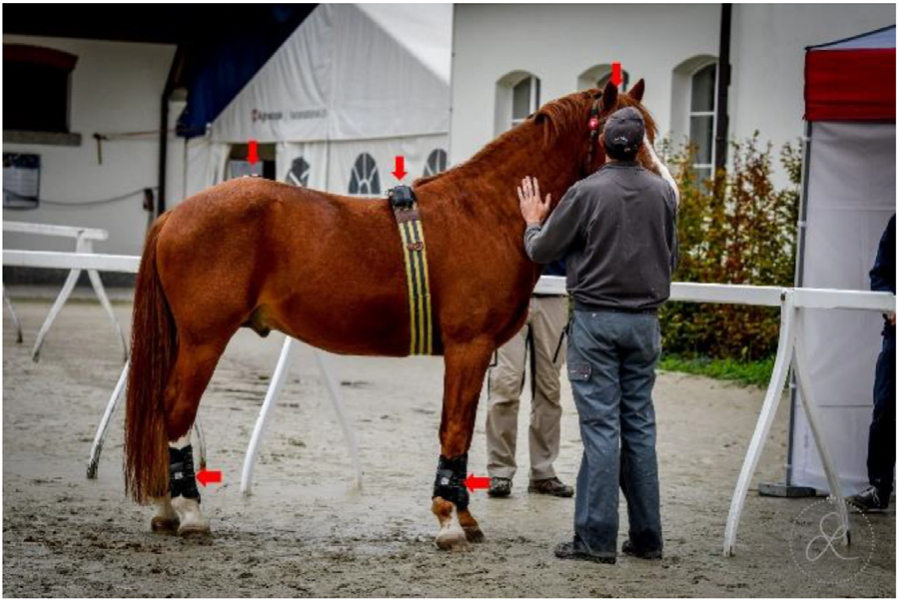
Horse equipped with the seven IMU sensors from EquiMoves® on the head, withers, sacrum, and limbs shown with red arrows.

**Fig. 4:**
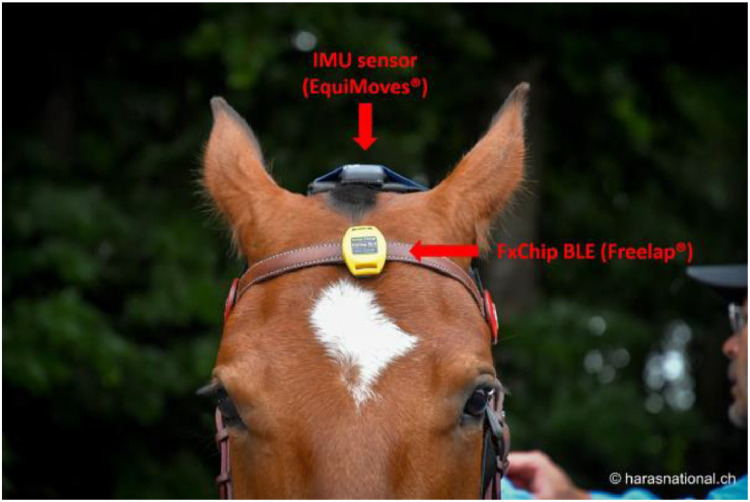
The IMU head sensor were fixed on a custom-made head piece with Velcro and the FxChip BLE to measure the time of a run was clipped to the browband of the bridle.

**Fig. 5:**
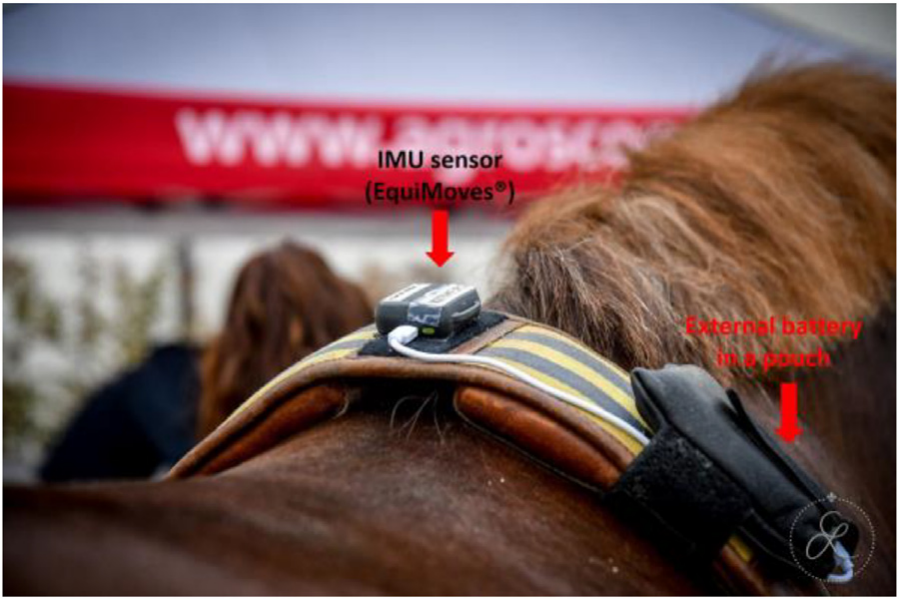
The girth was equipped with a portable USB battery for the withers sensor with the integrated GNSS-node to prolong battery life.

**Fig. 6:**
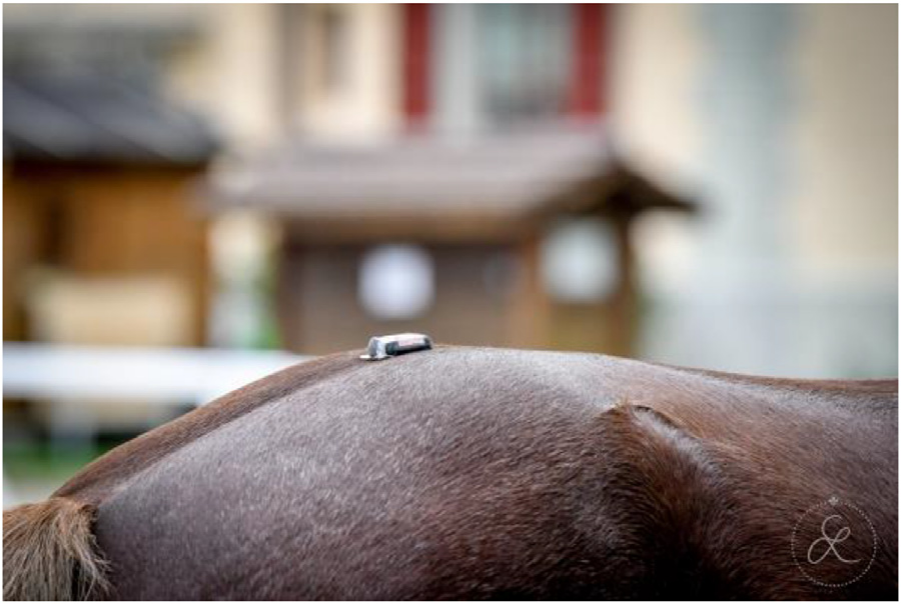
The sacrum sensor was placed on the tuber sacrale with double-sided sticky tape.

**Fig. 7:**
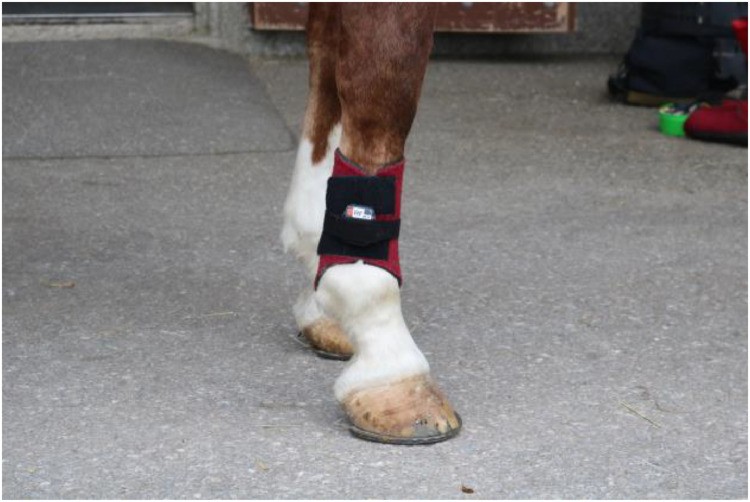
The limb sensors were fixed with Velcro sewn on the outside of the boot and secured with a double-sided Velcro strap around the boot.

**Fig. 8:**
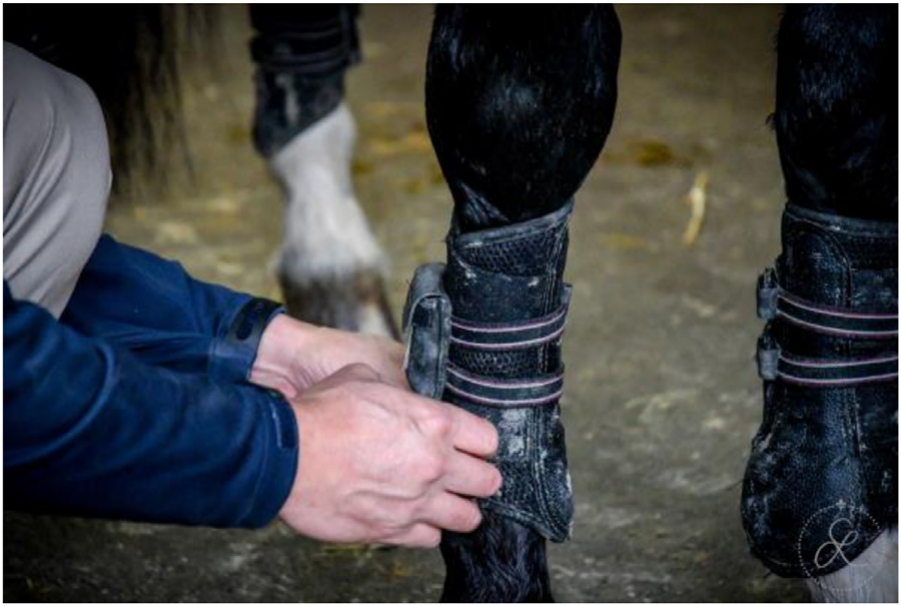
In case of rain, the IMU sensors were placed in leather pouches to protect them from water damage.

Horses were equipped with seven inertial measurement units (IMU) sensors (Promove-mini, Inertia Technology, The Netherlands) attached to the head, withers, sacrum, and lower limbs, as shown in Fig. 3. The head IMU was attached with Velcro to a custom-made padded poll guard fixed to the headpiece of the bridle (Fig. 4). The withers sensor with the integrated GNSS-node for speed measurement was mounted on a surcingle using Velcro (Fig. 5). The sacrum sensor was attached with double-sided tape (MED 6364R Avery Dennison Medical) over the tuber sacrale (Fig. 6). The limb sensors were fixed to customized boots using Velcro to the lateral aspect of the metacarpal/metatarsal bones (Fig. 7). In case of heavy rain, the limb sensors were wrapped in cling film (plastic wrap for food) and placed into custom-made leather pouches before fixing them around the boots (Fig. 8). For consistency, the height of the sensors on the metacarpus/metatarsus was fixed at a distance of three fingers above the palpable joint space of the metacarpophalangeal joint and four fingers above the joint space of the metatarsophalangeal joint. Obviously, this distance depended from the person instrumenting the horse (“Placer” in the metafile).

Sampling frequency of the IMU sensors was 200 Hz. The low-g accelerometer of the IMUs placed at the limbs had a measuring range of ±16 g and the high-g accelerometer a range of ±200 g; the low-g accelerometer of the IMUs placed at the upper body had a measuring range of ±8 g and the high-g accelerometer a range of ±100 g. The gyroscope of all IMUs was set to measure at 2000 °/sec. Each sensor was equipped with an internal memory to ensure there would be no data loss during the entire recording time. From 2021 onwards, the withers sensor was additionally equipped with a global positioning system node (GNSS) set to measure at 5 Hz. f.

In addition, each horse was fitted with a FxChip BLE from Freelap® to measure the time laps between the two Freelap® gateways (Tx Track Pro) positioned at the beginning and end of the runway. The chip on the browband of the bridle transmitted the time when the horse passed the gateway to the Freelap® application.

### 3.3 Gait measurement

Generally, the horse was first walked, then trotted along the runway, but very excited horses were trotted first to deal with excess energy, and then walked in a calmer state. An additional person (“Handler 2”) was present to encourage the horse if necessary (shaking a whip, clapping hands, clicking tongue). The horses were never encouraged excessively or touched with the whip.

**Fig. 9:**
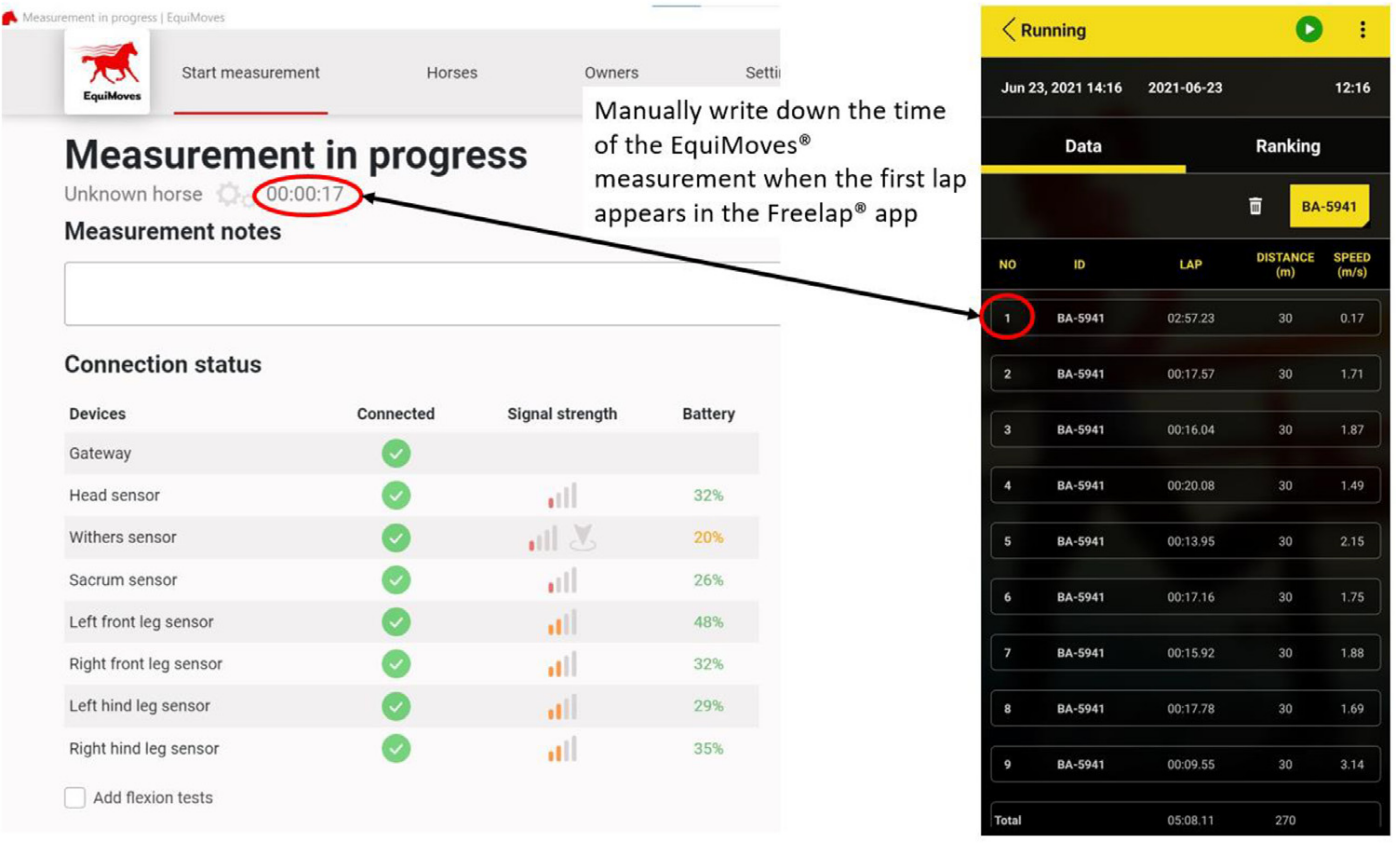
Screenshots of the EquiMoves® and Freelap® apps to illustrate how the measurements for each horse were timewise assigned.

The Freelap® and EquiMoves® systems were synchronized manually for each horse by recording the time the horse passed the first Freelap® gateway (first signal from Freelap®) to the timing of the measurement in EquiMoves® (“time to first signal” in the protocol sheet, Fig. 9). In case of overall technical failure by the Freelap® system, we extracted the speed from the EquiMoves® system or a stopwatch (see variable Speed_mes).

### 3.4 Data processing

After each measurement the data from the sensors was synchronized and processed using the EquiMoves® software (version 0.0.211001, released 14/10/2021). The EquiMoves® software used stride and gait detection algorithms described in [4,5], to split the measurements into segments depending on the gait and to extract stride by stride information. Furthermore, it filtered the data and evaluated upper and lower body variables with methods described in [6].

### 3.5 Criteria for a successful measurement

**Fig. 10:**
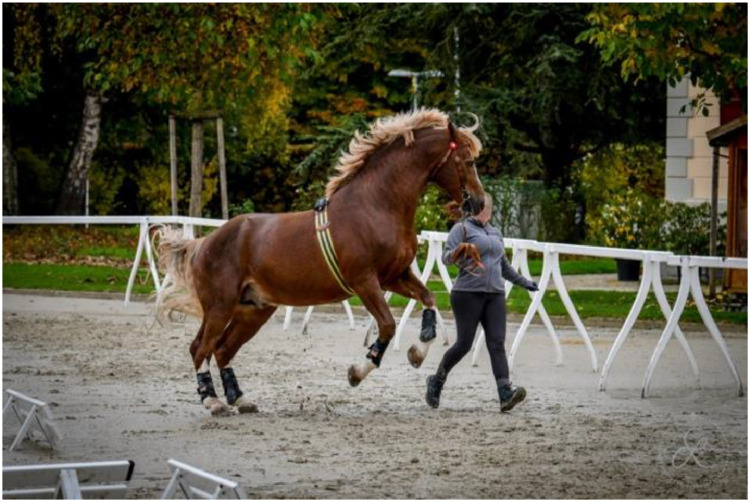
The horse had to stay at the same gait, without jumping, kicking out or shaking of head.

**Fig. 11:**
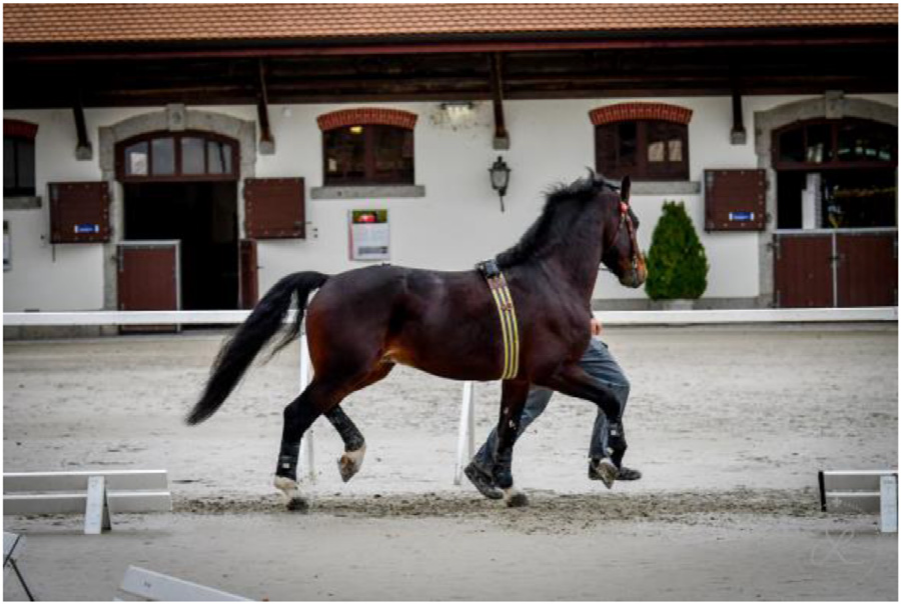
The handler should not pull the horse towards him as it affects the abduction-adduction angles and trunk posture.

A run was considered successful if the horse did not jump, stop or switch gaits, kept its head straight ahead without being influenced by the handler (Figs. 10 and 11), seemed at relative ease (neither unduly stressed, with short hurried steps, or breaking out in front, nor too slow, stumbling) and we ideally had timing information from Freelap®. Successful runs were noted in the protocol sheet, with an information on the timing linked to the Freelap® measurement for post-processing.

### 3.6 Data extraction and filtering

To facilitate a collective analysis of the data, the processed *.cbor* files from the EquiMoves® software were converted to *.json* files using a C++ based conversion algorithm processed in Microsoft Visual Studio (Visual Studio 2019, Microsoft) and imported into Matlab (Version R2023a, MathWorks). The imported data was already segmented into trials the same way they appeared in the EquiMoves® app. In addition, the hand-written trial notes from the field were manually transcribed into an .xlsx file and transferred into Matlab to correctly select the segments that were deemed as good trials from the EquiMoves® data. The speed of the selected trials was manually extracted from the Freelap® app and included in the trial notes. For each horse and gait, at least two successful runs as described above were selected.

Variables of interest from the selected trials were then extracted into one table. Additionally, further variables of interest were calculated with information from the field measurements, including: mean stride length (mean speed (Freelap)*mean stride duration), relative vertical ROM of the withers (withers ROM/withers height) and suspension duration (right: time of hoof-off (RF,LH) to time of hoof-on (LF,RH) and left: time of hoof-off (LF,RH) to time of hoof-on(RF,LH)). For the limb angles, the mean of the maximum angle for all selected trials was reported. For all other variables, the mean for all selected trial means was reported.

## Limitations

The major limitation was the limited access to some breeds compared to others, and that one breed (AR) had only been assessed on soft surface. Who placed the sensors and who walked/trotted the horse might also have influenced the measurement. We have therefore provided coded information on both of these factors in order that these effects can at least be mitigated statistically, but they tend to be confounded in the measurement place and date (e.g. there was only one placer for Arabians, another for Lusitanos). The precision of the timing of the segments, which would affect the mean stride length, stride frequency and speed was also difficult, as the extracted segmented data from the EquiMoves® software frequently contained a few acceleration and/-or deceleration strides outside the measuring range of the Freelap® speed measuring system. Due to field conditions, the runway was not always the same length. However, in the case of very short runways, the mean was calculated over several runs so that an adequate number of strides could be considered. Finally, the behaviour of the horse had a considerable impact on the number of strides useable for analysis.

## Ethics Statement

The experiments were run under the animal permit number VD3527b. No animal was harmed or unduly solicited over their coping capacity during the experiment. The exact location of the experiments is not provided to ensure anonymity of the horses and owners. The experiments complied with ARRIVE guidelines.

## CRediT authorship contribution statement

**Annik Imogen Gmel:** Conceptualization, Methodology, Formal analysis, Investigation, Data curation, Writing – original draft, Funding acquisition, Project administration. **Eyrún Halla Haraldsdóttir:** Conceptualization, Methodology, Formal analysis, Investigation, Data curation, Writing – review & editing. **Filipe Serra-Bragança:** Methodology, Software, Data curation. **Luis P Lamas:** Investigation. **Teresa V Rosa:** Investigation. **Monika Stefaniuk-Szmukier:** Investigation. **Weronika Klecel:** Investigation. **Markus Neuditschko:** Conceptualization, Writing – review & editing, Funding acquisition, Resources, Supervision, Project administration. **Michael A Weishaupt:** Conceptualization, Methodology, Investigation, Data curation, Writing – review & editing, Resources, Supervision.

## Data Availability

Kinematic data from owner-sound horses walking and trotting on a straight line (Original data) (Mendeley Data).
